# Longings of Pregnant Women

**Published:** 1846-03

**Authors:** 


*Longings of Pregnant Women.*—The appetites and longings of women in pregnancy are indubitable proofs of disordered blood; for, if we knew rightly how to trace effects to their causes, we should find, that the substances for which they long, contain the elements in which their blood is defective. Thus if a woman have a longing for cabbage, greens, radishes, or any species of the Brassica, we may know that sulphur and phosphorus, and most probably hydrogen and nitrogen, are deficient. If she have longing for fish,, we may conclude that gelatine and phosphorus are deficient. If she long for carbon in any shape, it is needed to neutralize the nitrogen and hydrogen in excess—and if she long for lime and magnesia, they are defective. In these matters, physiological observers have done little more than recount the fancies of gestating women, and leave them to the pity or ridicule of medical men. "As fanciful as a pregnant woman," is an old standing proverb.—*Dr. Shearman in N. Y. Jour. of Med. and Surg.*

